# Effect of Substrate Conductivity on the Transient Thermal Transport of Hygroscopic Droplets during Vapor Absorption

**DOI:** 10.3390/mi11020193

**Published:** 2020-02-13

**Authors:** Zhenying Wang, Daniel Orejon, Khellil Sefiane, Yasuyuki Takata

**Affiliations:** 1 International Institute for Carbon-Neutral Energy Research (WPI-I2CNER), Kyushu University, 744 Motooka, Nishi-ku, Fukuoka 819-0395, Japan; 2 Department of Mechanical Engineering, Thermofluid Physics Laboratory, Kyushu University, 744 Motooka, Nishi-ku, Fukuoka 819-0395, Japan; 3 Institute for Multiscale Thermofluids, School of Engineering, The University of Edinburgh, Edinburgh EH9 3FD, Scotland, UK; 4 Tianjin Key Lab of Refrigeration Technology, Tianjin University of Commerce, Tianjin 300134, China

**Keywords:** thermal effects, substrate conductivity, absorptive heating, evaporative cooling, vapor pressure difference

## Abstract

In all kinds of liquid desiccant dehumidification systems, the temperature increase of the desiccant solution due to the effect of absorptive heating is one of the main reasons of performance deterioration. In this study, we look into the thermal effects during vapor absorption into single hygroscopic liquid desiccant droplets. Specifically, the effect of substrate conductivity on the transient heat and mass transfer process is analyzed in detail. The relative strength of the thermal effect and the solutal effect on the rate of vapor absorption is investigated and compared to the thermal effect by evaporative cooling taking place in pure water droplets. In the case of liquid desiccants, results indicate that the high thermal conductivity of copper substrates ensures more efficient heat removal, and the temperature at the droplet surface decreases more rapidly than that on Polytetrafluoroethylene (PTFE) substrates. As a result, the initial rate of vapor absorption on copper substrates slightly outweighs that on PTFE substrates. Further analysis by decomposing the vapor pressure difference indicates that the variation of vapor pressure caused by the temperature change during vapor absorption is much weaker than that induced by the concentration change. The conclusions demonstrate that a simplified isothermal model can be applied to capture the main mechanisms during vapor absorption into hygroscopic droplets even though it is evidenced to be unreliable for droplet evaporation.

## 1. Introduction

Liquid desiccant is one type of aqueous salt solution characterized by its hygroscopic properties, and has been widely applied in various dehumidification and absorption systems [1,2]. Due to the existence of specific ions with strong adhesion to water molecules, the water vapor pressure at the droplet surface is reduced when compared to the partial vapor pressure of the surrounding air [3]. As a result, water vapor diffuses from the air side towards the liquid–air interface, and gets absorbed into the droplet [4]. Along with vapor–liquid phase change, the latent heat released will heat up the liquid solution, which is one of the main reasons of performance deterioration in all kinds of dehumidification devices [5].

Studies on the thermal effect taking place during the evaporation of sessile volatile droplets have been carried out extensively in the past years. The evaporation of volatile molecules cools down the droplet surface, and the effect of evaporative cooling is proved to strongly affect the evaporative mass flux. Typically, the thermal conductivity of the solid phase is several orders higher than that of the gas phase; therefore, heat conduction into the solid substrate plays an important role in the heat transfer process especially for droplets in still air with weak convection. Experiments carried out by Dunn et al. [6,7] confirm the strong effect of substrate conductivity on droplet evaporation, and an improved mathematical model is derived which relates the saturation vapor concentration at the droplet interface with the localized surface temperature. Sobac and Brutin [8] investigated the influence of substrate properties on the evaporation process in both hydrophilic and hydrophobic cases. Results highlight the need for more accurate models to account for the buoyant convection in vapor transport as well as the evaporative cooling and heat conduction between the droplet and the substrate [9]. Similar experiments were conducted by Talbot et al. [10] on picoliter droplets, which suggest that the thermal effects on the evaporation rate are much stronger for droplets on low-thermal-conductivity substrates than those on high-thermal-conductivity substrates. They also drew a similar conclusion that the evaporation time is underestimated by existing isothermal models. 

To compensate the weakness of the isothermal models, Sefiane et al. [11] proposed a general expression for droplet evaporation which accounts for the thermal effect associated with evaporative cooling and includes the effects of both substrate and liquid properties. Similar theoretical trials were also conducted subsequently by Xu and Ma [12]. Zhang et al. [13] established a mathematical model to account for the thermal effect in an evaporating pure liquid droplet. The results show the nonmonotonic distribution of interfacial temperature, which is further explained combining the effect of evaporative cooling and heat of conduction through the liquid and the substrate. By solving a similar model using a finite element method, Wang et al. [14] characterized the combined effects of the underlying substrate and evaporative cooling. Results show that the influence of substrate properties on the evaporation process also depends on the strength of evaporative cooling. Other experimental and numerical investigations on the thermal effect also include the influence of substrate heating [15], thermal Marangoni [16], heat flux distribution [17], etc.

The existing studies indicate that the thermal effect induced by interfacial phase change affects the spatiotemporal evolution of mass flux at the liquid–air interface. Opposite to droplet evaporation, the vapor absorption into hygroscopic solution droplets will induce a strong effect of absorptive heating. The thermal effect along with heat conduction governs the temperature distribution within both the liquid droplet and the solid substrate, which in turn affects the rate of vapor absorption. 

In our previous research, we investigated the mechanisms of droplet growth and spreading [4], as well as the effects of ambient temperature, humidity, and surface wettability on the vapor absorption process [18]. In this study, we investigate the thermal effects and demonstrate its relation with the substrate properties during vapor absorption into hygroscopic liquid desiccant droplets. Experiments are carried out for four representative environmental conditions, where the evolution of droplet profile and the temperature distribution at the droplet surface are extracted using optical imaging and infrared (IR) thermography. Results on substrates with different thermal conductivity and controlled wettability indicate the strong effect of substrate properties on the spatial-temporal evolution of interfacial temperature and mass flux. The relative strength of thermal effect on the transient heat transfer and on the air-side vapor diffusion during evaporation and vapor absorption are compared and summarized.

## 2. Materials and Methods

**Figure 1:**
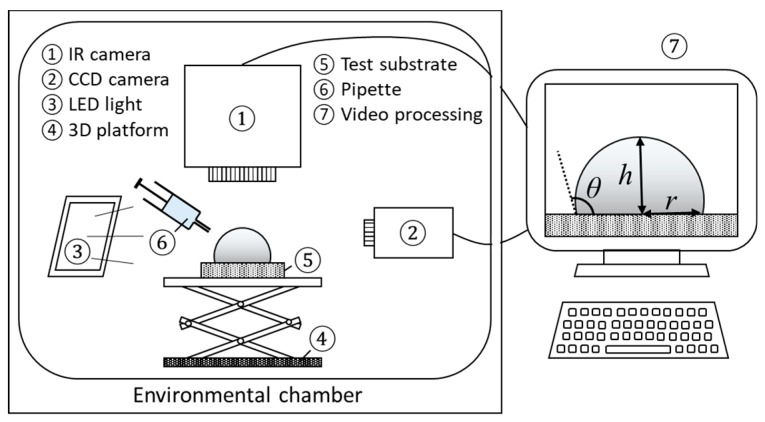
Overview of the experimental setup, including environmental chamber, charge-coupled device (CCD) camera, IR camera, back light, stainless steel vertical platform, droplet dosing system, and data acquisition system with ImageJ^®^ and Matlab^®^.

Experiments are conducted within an environmental chamber with accurately controlled conditions (800 L, −20–100 °C, 20–98% *RH*, PR-3KT from ESPEC Corp., Osaka, Japan). The accuracy of the temperature control is reported to be ± 0.5 °C, while the accuracy of the humidity control is ± 5% *RH*. Shown in Figure 1, during experiments, the evolution of droplet profile is recorded with a high-definition charge-coupled device (CCD) camera (Sentech STC-MC152USB with a RICOH lens and 25-mm spacing ring from OMRON SENTECH Corp., Kanagawa, Japan) at 4.8 fps, while a LED backlight is applied to enhance the image contrast. An IR camera, FLIR SC-4000 (Wilsonville, OR, USA), with a spectral range between 3.0 and 5.0 µm and a resolution of 18 mK, is set up vertically looking at the substrate and the deposited droplet from the top. The temperature evolution at the droplet liquid–gas interface is then recorded at 2 fps. Videos are subsequently processed with external software and self-developed programs, such as ImageJ^®^ and Matlab^®^.

Polytetrafluoroethylene (PTFE) and copper, two types of commonly used packing materials in dehumidification systems, are applied as the testing substrates [1]. The dimensions of both copper and PTFE substrates are 20 mm × 20 mm × 10 mm, where the height is 10 mm. To rule out the influence of surface wettability on the droplet behavior, a uniform fluorinated ethylene propylene (FEP) coating layer is deposited onto both substrates following the same self-assembled monolayer (SAM) procedure. Since the thickness of the FEP coating is the same for both substrates and can be considered negligible when compared to the bulk material (thickness of the SAM is in the order of nanometers while the thickness of the studied samples is 10 mm), the effect of thermal conductivity of PTFE and copper can be investigated for droplets with similar contact angles of 106 ± 3°. 54 wt. % LiBr-H_2_O solution from Sigma-Aldrich is used as the testing fluid for vapor absorption experiments, and the droplet volume is controlled as 2.5 ± 0.3 μL. Contrast experiments of droplet evaporation are conducted using distilled water (Sigma-Aldrich). Other detailed properties of the testing fluids and substrates are listed in Table 1 and Table 2.

Before experiments, the substrate samples are cleaned using an ultrasonic bath and deionized water, and are further dried with filtered compressed air to remove any possible remaining dusts or contaminants. After that, the testing fluid and substrate are placed inside the environmental chamber for more than 30 min until thermal-equilibrium state is attained. Then, a droplet with a controlled volume is deposited gently onto the substrate and then real-time recording of both CCD camera and IR camera is triggered. To ensure the reliability of the experimental results, each experiment is repeated 5 times. We note here that, since the characteristic length of the droplet lies below the capillary length ($\lambda =\sqrt{\gamma_{\mathrm{lg}}/\rho g}$, *ca.* 2.7 mm for water and *ca.* 2.42 mm for 54 wt.% LiBr-H_2_O solution at 20 °C), we assume the droplet shape as a spherical cap and derive the droplet volume and other parameters accordingly.

## 3. Results and Discussion

**Figure 2:**
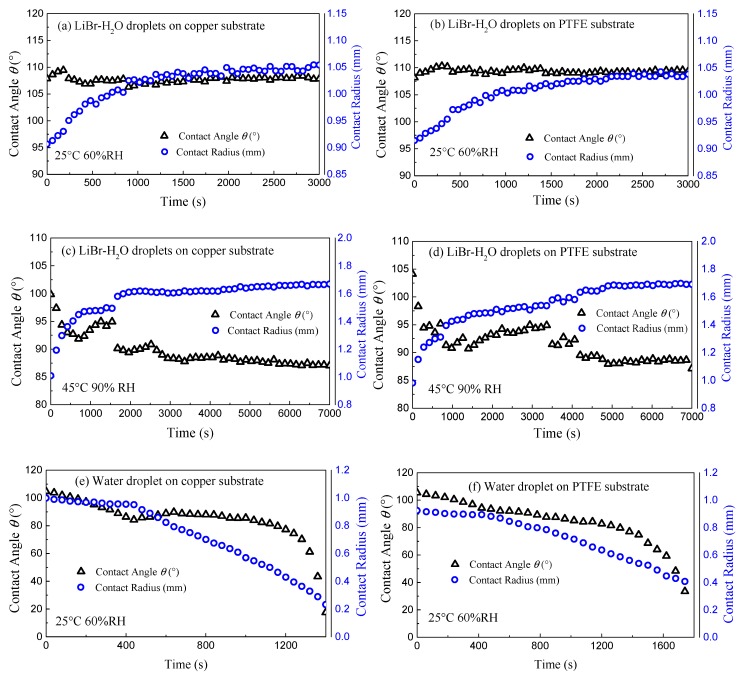
Evolution of contact angle *θ* (black triangles points) and contact radius *r* (blue circles points) of LiBr-H_2_O (**a**–**d**) and pure water droplets (**e,f**) on copper (**a,c,e**) and PTFE (**b,d,f**) substrates coated with FEP for 25 °C—60% *RH* (**a,b,e,f**) and 45 °C—90% *RH* (**c,d**) conditions.

Figure 2 indicates the representative varying curves of contact angle *θ* and contact radius *r* of LiBr-H_2_O droplets for 25 °C—60% *RH* and 45 °C—90% *RH* conditions, as well as the contrast pure water droplets for 25 °C—60% *RH* conditions on copper and PTFE substrates. 

Due to the existence of uniform FEP coating, the evolution of contact angle and contact radius follows similar qualitative trend regardless of the substrate conductivity. For LiBr-H_2_O droplets, at 25 °C and 60% *RH*, the contact angle increases slightly at first, then decreases until reaching fully equilibrium state, and stays constant during the remaining of the vapor absorption experiment. The initial increase of contact angle is due to fast vapor absorption, in which case the advancing contact line alone cannot keep up with the rapid volume expansion. As vapor absorption goes on, the solute concentration decreases due to water uptake, which causes the decrease in the liquid–gas surface tension and the rate of vapor absorption. As a joint result of decreasing surface tension and absorption rate, the contact angle decreases in the later stage. At the same time, the contact radius increases continuously following a saturation trend until the droplet reaches equilibrium with the ambient as presented in Figure 2a,b. In the case of 45 °C and 90% *RH*, upon deposition, the droplet contact angle is lower than that for 25 °C as a consequence of the lower surface tension of the liquid desiccant at higher temperature. Then, the contact angle decreases quickly at the initial moment. Along with vapor absorption, the contact angle fluctuates with the advancing stick-slip behaviors of the droplet (Figure 2c,d) [18] until both radius and contact angle show a plateau at which equilibrium is reached with the ambient. 

For pure water droplets, the evolution of contact angle and contact radius follows the typical trend of an evaporating volatile droplet on a hydrophobic substrate [19,20]. At the initial period, the contact line remains pinned with decreasing contact angle, i.e., constant contact radius (CCR) mode. Then, the contact line starts to recede and the contact angle keeps constant, i.e., constant contact angle (CCA) mode. At the final stage, the small droplet diminishes with the contact angle and contact radius decreasing simultaneously as in the mixed mode.

**Figure 3:**
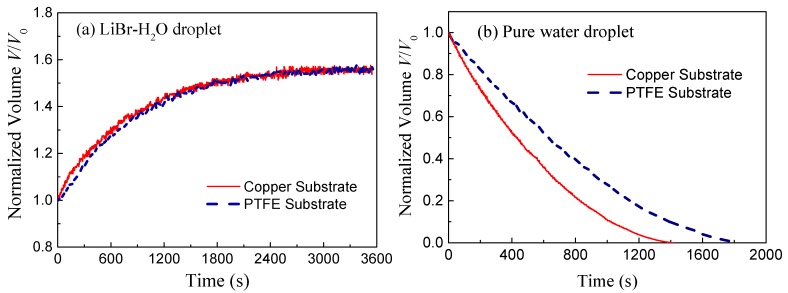
Evolution of normalized volume for (**a**) LiBr-H_2_O droplets and (**b**) pure water droplets on copper (solid red lines) and PTFE (dashed blue lines) substrates coated with FEP along with time for 25 °C and 60% *RH* conditions.

Figure 3 shows the variation of normalized volume *V*/*V*_0_ of LiBr-H_2_O droplets and pure water droplets on copper and PTFE substrates taking 25 °C and 60% *RH* conditions as a representative example. At this ambient condition, the rate of vapor absorption is moderate while the vapor absorption phenomenon is apparent and the equilibrium state is easily reached. Due to vapor absorption, the volume of LiBr-H_2_O droplets increases rapidly at first and then slows down as the droplet gets saturated with water. Comparing the increasing trend of the two curves, it can be seen that the rate of vapor absorption of droplets on copper substrates slightly outweighs that of droplets on PTFE substrates at the initial period (0–600s), while at the final equilibrium stage, the droplet volume attained in the two cases is the same. Differently, for pure water droplets, the evaporation rate is found to be greatly affected by the substrate conductivity. As shown in Figure 3b, water droplets on copper substrates exhibit an apparently higher evaporation rate and shorter lifetime than those on PTFE substrates, which corresponds with the results in previous studies [10,11].

**Figure 4:**
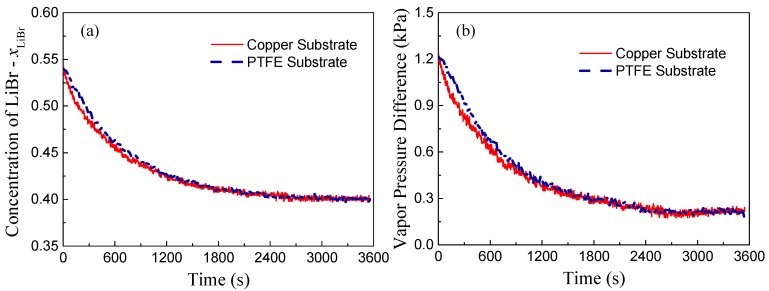
Evolution of (**a**) solute concentration, *x*_LiBr_, and (**b**) vapor pressure difference between surrounding air and liquid-air interface of LiBr-H_2_O droplets on copper and PTFE substrates coated with FEP for 25 °C and 60% *RH* conditions.

Based on mass conservation, the solute concentration inside the LiBr-H_2_O droplets (Figure 4a) can be calculated as established in the work of Wang et al. [4]. In addition, the vapor pressure difference between the ambient and the droplet surface (Fig. 4(b)) can be evaluated according to the fitting correlations derived by Patek et al. [21] and further implemented by Wang et al. for the absorption of LiBr-H_2_O liquid desiccant droplets [4]:

(1) $$\Delta P=P_{\mathrm{vapor},\mathrm{ambient}}-P_{\mathrm{vapor},\mathrm{surface}},\;P_{\mathrm{vapor},\mathrm{surfac}e}=P_{\mathrm{sat}}\left(\Theta \right),$$

where *P*_vapor,ambient_ is the partial vapor pressure of ambient air, *P*_vapor,surface_ is the vapor pressure at the liquid–air interface, and *P*_sat_ is the saturation vapor pressure of pure water at shifted temperature, Θ. Θ is function of the mole fraction, *x*_mole_, and temperature, *T*, of the LiBr-H_2_O solution, and can be calculated as Equation (2):

(2) $$\Theta =T-\sum_{i=1}^{8}a_{i}{\left(x_{\mathrm{mole}}\right)}^{m_{i}}{\left|0.4-x_{\mathrm{mole}}\right|}^{n_{i}}{\left(\frac{T}{T_{c}}\right)}^{t_{i}},$$

where *T*_c_ is the critical temperature of pure water, 647.096 K, *x*_mole_ is the mole fraction, *a* = {−2.41303 × 10^2^, 1.91750 × 10^7^, −1.75521 ×10^8^, 3.25432 × 10^7^, 3.92571 × 10^2^, −2.12626 × 10^3^, 1.85127 × 10^8^, 1.91216 × 10^3^}, *m* = {3, 4, 4, 8, 1, 1, 4, 6}, *n* = {0, 5, 6, 3, 0, 2, 6, 0}, and *t* = {0, 0, 0, 0, 1, 1, 1, 1}.

Calculation results indicate that the vapor pressure difference between the ambient and the droplet surface decreases along with time (Figure 4b), and since the driving force for vapor diffusion decreases, the rate of vapor absorption decreases accordingly. Different from other parameters, the substrate conductivity mainly affects the transient heat and mass transfer process. Therefore, despite the slight difference in the rate of vapor absorption during the initial non-equilibrium period, droplets on copper and PTFE substrates will finally reach the same state, i.e., temperature and solute concentration, with the same final volume for a given specific environmental condition; reaching thermal, chemical, and thermodynamic balance with the environment.

**Figure 5:**
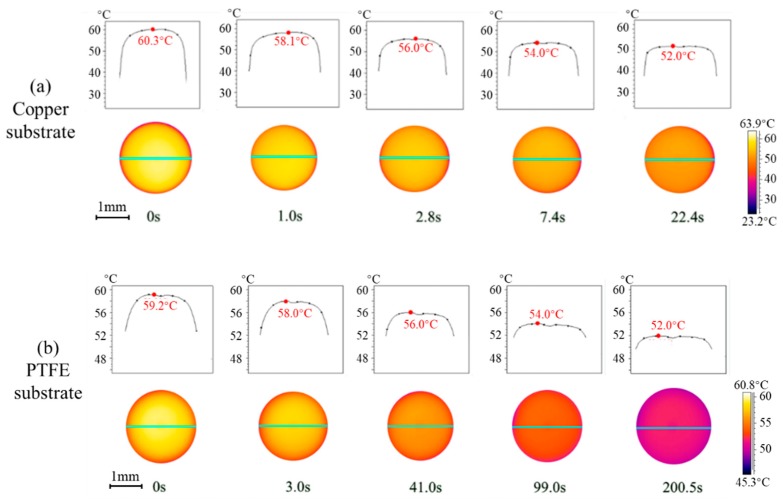
Spatiotemporal evolution of interfacial temperature of LiBr-H_2_O droplets on (**a**) copper and (**b**) PTFE substrates coated with FEP.

To provide more fundamentals and insights on the transient heat transfer during vapor absorption, the interfacial temperature of droplets on copper and PTFE substrates is investigated by IR thermography. Figure 5 shows the experimental results at 45 °C and 90% *RH* as the rate of vapor absorption is apparently high allowing for easier comparison. The spatial temperature distribution across the LiBr-H_2_O droplet is overall homogenous throughout the vapor absorption process, indicating a negligible thermal Marangoni effect at the surface. The droplet surface experiences the highest temperature at the initial moment due to fast vapor absorption which starts right after the droplet is generated from the needle and contacts the humid air. After being deposited on the substrate, the absorbed heat is dissipated across the liquid droplet and into the solid substrate, and the interfacial temperature decreases along with time.

For droplets on the copper substrate (Figure 5a), it takes about 22 s for the interfacial temperature to decrease from the initial *ca.* 60 °C to *ca.* 52 °C, while on PTFE substrates (Figure 5b) it takes much longer, about 200 s from the initial *ca.* 59 °C to *ca.* 52 °C. The heat of conduction within the droplet and across the substrate can be evaluated by the characteristic time, expressed as, τ^*^ = ρc_p_*h*^2^/*k*, where *h* is the characteristic length, i.e., the height of the droplet or the thickness of the substrate. By making use of the characteristic time τ^*^, the time scale for heat conduction within the droplet is ~10 s, ~1 s for heat conduction across the copper substrate, and 10^2^–10^3^ s for heat conduction across the PTFE substrate. Results on the characteristic time for the heat of conduction agree with the experimental observations, and verify the dominating influence of substrate conductivity in the transient heat transfer and evolution of interfacial temperature of the droplet during vapor absorption.

**Figure 6:**
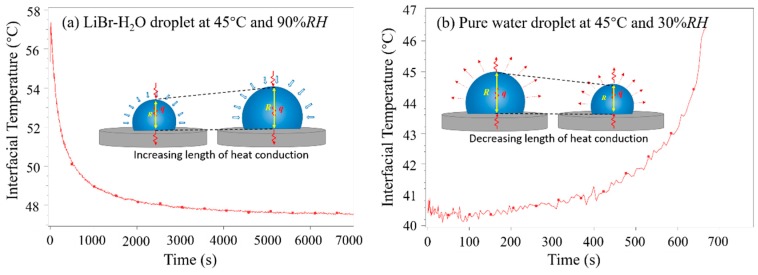
Variation of average interfacial temperature of (**a**) a LiBr-H_2_O droplets at 45 °C and 90% *RH*, and (**b**) a pure water droplet at 45 °C and 30% *RH* on PTFE substrates coated with FEP.

Figure 6 indicates the representative varying curves of average interfacial temperature of LiBr-H_2_O droplets (Figure 6a) and that of pure water droplets (Figure 6b) on a PTFE substrate. For water droplets (Figure 6b), the interfacial temperature is low at the initial period as a result of evaporative cooling, and then increases due to heat supply from the substrate. Moreover, the increase of interfacial temperature speeds up towards the end of the droplet lifetime. This is because, as water evaporates, the volume of water droplet shrinks. In this case, the length for heat conduction from the liquid–solid interface to the liquid–air interface decreases and the heat capacity of the liquid droplet become smaller; therefore, the droplet surface warms up quickly. By comparison, for LiBr-H_2_O droplets (Figure 6a), the decrease of interfacial temperature slows down along with time. This is because, as vapor absorption goes on, the volume expansion of the LiBr-H_2_O droplet causes an increase in both the characteristic length for heat conduction and the heat capacity of the droplet. In this case, the thermal resistance for heat conduction increases, and the temperature decrease at the droplet surface slows down.

**Figure 7:**
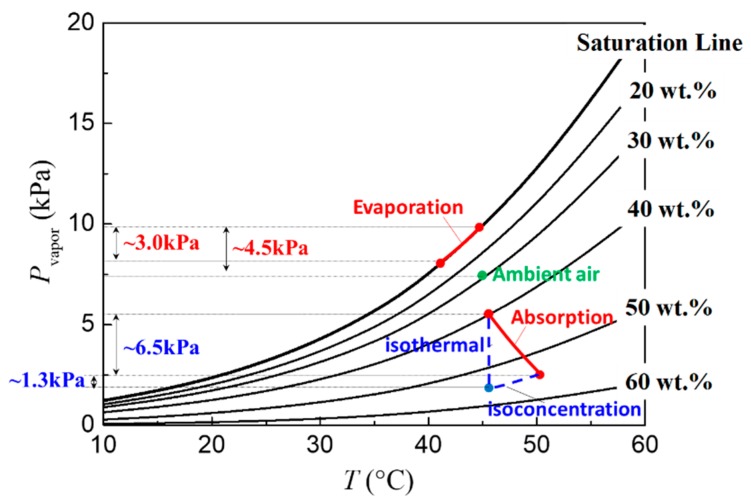
Evolution of water vapor pressure *P*_vapor_ along with interfacial temperature for pure water droplets during evaporation and LiBr-H_2_O droplets with different solute concentrations during vapor absorption showed in red lines.

Next, we discuss the evolution of water vapor pressure at the droplet surface. Figure 7 shows the evolution curves as function of interfacial temperature in the case of pure water droplets (saturation line) and LiBr-H_2_O droplets with different solute concentrations (20 wt.%–60 wt.%), which are calculated according to the *P*-*T*-*x* correlations by Patek et al. [21] expressed in Equations (1) and (2). For water droplets, the effect of evaporative cooling will cause a decrease in the interfacial temperature, and therefore a decrease in the saturation vapor pressure at the droplet surface. Taking the condition of 45 °C and 60% *RH* as an example (for more clear demonstration), the temperature drop induced by evaporative cooling will bring about ~3.0 kPa decrease in the water vapor pressure at the liquid–air interface of pure water droplet, while the overall vapor pressure difference to drive vapor diffusion is only about 4.5 kPa by making use of the isothermal assumption. This indicates that the thermal effect cannot be neglected during droplet evaporation, and the isothermal model is no longer reliable [7,11]. On copper substrates, the high thermal conductivity ensures more efficient heat supply from the substrate so that the interfacial temperature is kept high. In this case, the water vapor pressure at the droplet surface is high, and the evaporation mass flux is large; therefore, the droplet lifetime is shorter than that on a PTFE substrate (Figure 3b). 

In the case of 54 wt. % LiBr-H_2_O droplets, the vapor absorption will induce the variation of both temperature and concentration as indicated in Figure 7. By dividing the vapor absorption process into an isoconcentration process (where the temperature changes alone) and an isothermal process (where the solute concentration changes alone), it can be seen that the variation of water vapor pressure induced purely by temperature change is about 1.3 kPa, while the variation induced by concentration change is about 6.5 kPa. This demonstrates that the vapor diffusion in the gas phase and the rate of vapor absorption is mainly controlled by the concentration variations of LiBr–H_2_O droplets instead of the interfacial temperature. Since the substrate conductivity only affects the transient heat transfer and the evolution of interfacial temperature, its effect on the rate of vapor absorption is therefore not apparent as evidenced by our experiments. This reminds us that, in the mathematical modeling of vapor absorption, more efforts must be made into the accurate description of the dominating mass transfer process where the concentration distribution is of high importance, while the thermal transport process can be properly simplified in order to improve the simulation efficiency, i.e., computing time.

## 4. Conclusions

This paper investigates the thermal effects along with vapor absorption into hygroscopic liquid desiccant droplets. The effect of substrate conductivity on the transient heat transfer process and on the rate of vapor absorption is investigated by experiments and theoretical analyses. Results indicate that substrate conductivity plays a crucial role in the transient heat transfer process as demonstrated by the more rapid decrease on the surface temperature of LiBr-H_2_O droplets on high-thermal conductivity copper substrates due to efficient heat removal. As a result of the thermal effect, droplets on copper substrates show a slightly higher rate of vapor absorption than those on low-thermal conductivity PTFE substrates. Further analyses by decomposing the variation of water vapor pressure indicate that, compared to the influence of temperature change, the water vapor pressure at the droplet surface is greatly affected by the change of solute concentration during vapor absorption, and therefore the rate of vapor absorption. We conclude that, even though the thermal effect cannot be neglected in the simulation of droplet evaporation as revealed by previous researchers, in the mathematical modelling of the vapor absorption process, the thermal effect can be properly simplified in order to improve the calculation efficiency, and more efforts should be put into accurately capturing the solute diffusion and convection within the droplet.

## Figures and Tables

**Table 1:** Properties of 54 wt. % LiBr solution and distilled water as: specific heat capacity *c*_p_ (kJ/kg/K); density *ρ* (kg/m^3^); liquid-gas surface tension *γ*_lg_ (mN/m); viscosity *υ* (mPa·s); thermal conductivity *k* (W/m/K); and saturation temperature *T*_sat_ (°C). Properties shown were obtained at 20 °C and at 1 atm.

Liquid Type	*c*_p_ (kJ/kg/K)	*ρ* (kg/m^3^)	*γ*_lg_ (mN/m)	*υ* (mPa·s)	*k* (W/m/K)	*T*_sat_ (°C)
54 wt.% LiBr solution	1.98	1600	91.54	4.751	0.4286	141
Distilled water	4.18	998	72.75	1.005	0.5984	100

**Table 2:** Properties of Polytetrafluoroethylene (PTFE) and copper substrates as density *ρ* (kg/m^3^); specific heat capacity *c_p_*(kJ/kg/K); thermal conductivity *k* (W/m/K); thermal diffusivity *α* (m^2^/s), $\alpha =k/\rho c_{p}$; and thickness *δ* (mm) at 20 °C and 1 atm. We state here that thermal diffusivities *α* in this table are the right values compared to the values earlier reported [4,18].

Material	*ρ* (kg/m^3^)	*c*_p_ (kJ/kg/K)	*k* (W/m/K)	*α* (mm^2^/s)	*δ* (mm)
PTFE	2200	1.05	0.25	0.108	10.0
Copper	8960	0.39	397	114	10.0
